# Taxonomic identification of bile salt hydrolase‐encoding lactobacilli: Modulation of the enterohepatic bile acid profile

**DOI:** 10.1002/imt2.128

**Published:** 2023-07-16

**Authors:** Ziwei Song, Shuo Feng, Xingchen Zhou, Zhengxing Song, Jing Li, Ping Li

**Affiliations:** ^1^ State Key Laboratory of Natural Medicines China Pharmaceutical University Nanjing China; ^2^ School of Life Science and Technology China Pharmaceutical University Nanjing China; ^3^ Beijing Key Laboratory of New Molecular Diagnosis Technologies for Infectious Disease, Department of Biotechnology Beijing Institute of Radiation Medicine Beijing China

**Keywords:** bile acid, bile salt hydrolase, gut microbiota, lactobacilli, phylotype

## Abstract

Bile salt hydrolases (BSHs) are enzymes that are essential for the enterohepatic metabolism of bile acids (BAs). BSHs catalyze the production of unconjugated BAs and regulate the homeostasis of BA pool. This study identified *Lactobacillus* as a crucial BSH‐encoding genus, and 16 main species were obtained using metagenomic data from publicly available human gut microbiome databases. Then, the 16 species of lactobacilli were classified into four typical categories by BSH phylotypes, including five species encoding BSH‐T0, six species encoding BSH‐T2, four species encoding BSH‐T3, and *Ligilactobacillus salivarius* encoding both BSH‐T0 and BSH‐T3. The lactobacilli with the highest in vitro deconjugation activities against seven conjugated BAs were the BSH‐T3‐encoding strains. Furthermore, in vivo studies in mice administered four representative lactobacilli strains encoding different BSH phylotypes showed that treatment with BSH‐T3‐encoding *Limosilactobacillus reuteri* altered the structure of the gut microbiome and metabolome and significantly increased the levels of unconjugated BAs and total BA excretion. Our findings facilitated the taxonomic identification of crucial BSH‐encoding lactobacilli in human gut microbiota and shed light on their contributions toward modulation of the enterohepatic circulation of BAs, which will contribute to future therapeutic applications of BSH‐encoding probiotics to improve human health.

## Highlights

- *Lactobacillus* was identified as a crucial genus encoding bile salt hydrolase (BSH), and 16 main species of lactobacilli were classified into four categories according to BSH phylotypes.
- BSH‐T3‐encoding strains had the highest in vitro deconjugation activities.
- Treatment with BSH‐T3‐encoding *Limosilactobacillus reuteri* altered the enterohepatic bile acid profile in mice.

## Graphical abstract

Based on our previous work, *Lactobacillus* was identified as a crucial genus encoding bile salt hydrolase (BSH). Sixteen species of lactobacilli were obtained and classified into four typical categories according to BSH phylotypes used in silico approaches. Then, an in vitro detection experiment showed that the deconjugation ability of the lactobacilli strains could be represented as follows: Lac (BSH‐T3) ≈ *Ligilactobacillus. salivarius* > Lac (BSH‐T2) > Lac (BSH‐T0). Furthermore, in vivo studies of four representative strains from four categories showed that administration of *Limosilactobacillus reuteri* altered the structure of the gut microbiome and metabolome in mice and modulated the enterohepatic circulation of bile acids (BAs), specifically increasing total BAs and some unconjugated BAs.

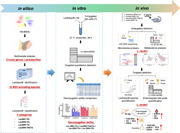

## INTRODUCTION

Bile acids (BAs) are initially synthesized from cholesterol in the hepatocytes and then covalently attached to glycine or taurine and stored in the gallbladder. In response to dietary intake, conjugated BAs in the gallbladder are transferred to the gut to generate unconjugated BAs and secondary BAs via catalysis by a series of enzymes in the gut microbiota. Approximately 95% of all BAs (including primary and secondary BAs; conjugated and unconjugated BAs) are reabsorbed by the intestine to enter the liver through the portal vein, and the rest are excreted in feces [1]. Moreover, the metabolic characteristics enable the formation of a dynamic BA pool that facilitates enterohepatic circulation in the body [2]. These metabolic processes are under negative feedback control through activation of the nuclear receptor farnesoid X receptor in the ileum and liver [3].

Bile salt hydrolases (BSHs) in gut microbiota are responsible for cleaving the amide bonds in conjugated BAs and increasing the complexity of the BA pool [4]. The unconjugated BAs produced by BSHs could act as signaling molecules that not only regulate BA metabolism and transport but also facilitate various metabolic activities that are critical for balancing lipid and glucose metabolism [5, 6, 7, 8], insulin sensitivity [9, 10], and innate immunity [11, 12] in the body. Disruption of the hepatic BA pool homeostasis is likely to lead to the development of cholestasis [13] and several other liver diseases, including nonalcoholic fatty liver disease [14, 15], liver cancer [16], and cholangiocarcinoma [17, 18]. Impaired BA homeostasis can lead to gastrointestinal diseases, such as irritable bowel syndrome [19], inflammatory bowel disease [12], and colorectal cancer [20]. Earlier studies on BSHs in bacteria focused on the identification and purification of BSHs from different bacteria [21, 22], screening of strains showing BSH activity in vitro [23, 24], and discovery of novel BSHs [25]. Recently, due to technological advancements and the pursuit of precision therapeutics, research studies have begun to consider the segmentation of BSHs and the functional differences in host metabolism. Jia et al. [26] collected 5790 BSH homologs and classified these into seven clusters based on a sequence similarity network. In our previous study, BSH‐encoding strains were systematically identified and classified into eight phylotypes based on phylogenetic analyses, with different specific activities in vitro [27]. Parasar et al. [28] revealed through chemoproteomic analyses that the relative contributions of individual BSHs to fecal BA levels were varied in a murine model of inflammatory bowel disease.

Among BSH‐encoding bacteria, *Lactobacillus* has been reported to contribute to the majority of the total BSH activity in vivo [29]. Our previous work provided similar evidence that BSH‐T3, encoded only by *Lactobacillus*, had the highest specific activity. However, *Lactobacillus* also encodes BSHs that belong to other phylotypes, suggesting differences in activity [27]. A study on BSHs repertoire in lactobacilli across 170 different species also speculated that nonhomologous BSH genes would show differences with respect to activity in vivo [30]. Therefore, further studies on taxonomic classification and functional evaluation of BSH‐encoding lactobacilli are necessary. In addition, the genus *Lactobacillus* was reclassified into 25 genera based on a polyphasic approach, including the emended genus *Lactobacillus* [31].

Based on our previous findings that BSHs were distributed in 591 strains within 117 genera of the human microbiota [27], an in‐depth analysis of these BSH‐encoding bacteria was conducted to (1) reveal crucial BSH‐encoding genus in human gut microbiota; (2) classify the BSH‐encoding lactobacilli according to a phylogenetic‐based system and assess their deconjugation activity in vitro; and (3) investigate the effects of administration of representative lactobacilli strains on the structure of the gut microbiome, metabolome, and enterohepatic BA pools in mice.

## RESULTS

### Characteristics of bacteria encoding BSH

**Figure 1:**
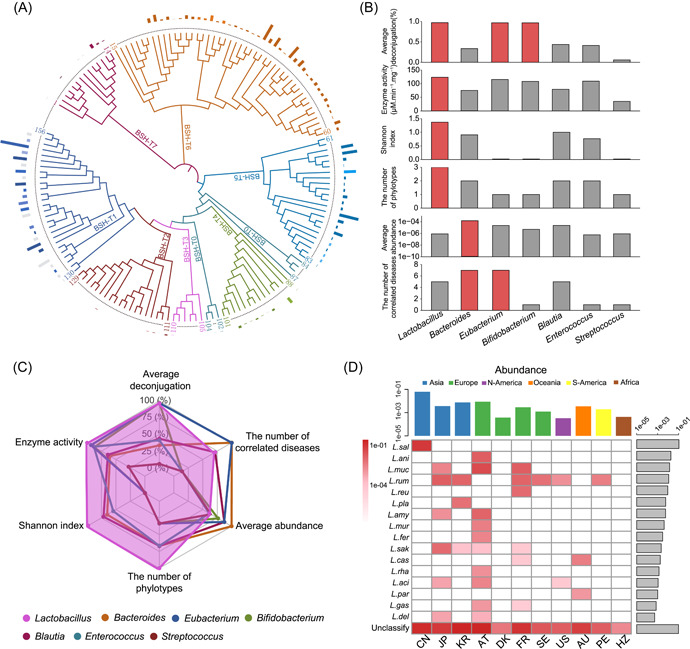
Comprehensive analysis of bile salt hydrolase (BSH)‐encoding bacteria. (A) Phylogenetic tree of 156 BSHs, accompanied by their relative abundance in the human microbiome. Columns with the same color represent BSHs from the same genus. In the same BSH phylotype, the lighter the color of the column, the lower the proportion of the total relative abundance of BSHs encoded by the genus. Genera with abundance of less than 5% are represented by gray columns. (B) Multiple factor analysis of representative BSH‐encoding genera. The diversity is represented by the Shannon index, the deconjugation ability is defined as the proportion of 100 μM conjugated bile salts hydrolyzed to unconjugated bile salt by BSHs after 48 h of reaction (detected by liquid chromatography triple quadrupole mass spectrometry), and the specific enzymatic activity is determined by the amount of BSHs that release 1 μmol glycine or taurine from 20 mM conjugated bile salts per minute in ninhydrin assay. The red columns represent the top genera during the analysis of the corresponding factor. (C) Radar analysis of representative BSH‐encoding genera; the data for each factor were normalized. (D) Relative abundance of BSH‐encoding lactobacilli strains in different populations.

A phylogenetic tree of the eight BSH phylotypes (including 156 BSHs from 90 species) was constructed and the abundance in human gut microbiome based on previous results was determined (Supporting Information: Figure S1) [27]. The most abundant genera among the eight phylotypes included *Enterococcus* (18.32% of the total relative abundance of BSH‐T0), *Eubacterium* (18.53% of the total relative abundance of BSH‐T1), *Streptococcus* (43.96% of the total relative abundance of BSH‐T2), *Lactobacillus* (before the taxonomic reorganization of lactobacilli in 2020 [31]; 100% of the total relative abundance of BSH‐T3), *Bifidobacterium* (50.97% of the total relative abundance of BSH‐T4), *Bacteroides* (82.13% and 89.89% of the total relative abundance of BSH‐T5 and BSH‐T6, respectively), and *Blautia* (73.14% of the total relative abundance of BSH‐T7; Figure 1A, Supporting Information: Figure S1). A multivariate analysis was performed on the seven genera based on the following: abundance, diversity (Shannon index), deconjugation ability (the proportion of 100 μM conjugated bile salts hydrolyzed to unconjugated bile salt by BSHs after 48 h of reaction, detected by liquid chromatography triple quadrupole mass spectrometry), specific activity (the amount of BSHs that release 1 μmol glycine or taurine from 20 mM conjugated bile salts per minute in a ninhydrin assay), number of encoded BSH phylotypes, and number of related diseases (Figure 1B) [27]. The results showed that the BSH‐encoding lactobacilli strains had the highest average deconjugation (97.13%), specific activity (124 µM min^−1^ mg^−1^), Shannon index (1.4), and number of phylotypes (3 out of 7). The radar chart clearly showed that *Lactobacillus* was the most critical genus among the representative BSH‐encoding genera (Figure 1C, Supporting Information: Figure S2).

### Lactobacilli species encoding BSH in human gut microbiome

Genomic sequences of 581 strains from 166 species of lactobacilli were downloaded from the Ensembl database (Supporting Information: Table S1). Then, 25 human metagenome‐origin lactobacilli species were obtained based on the Human Metagenome Project (HMP) database (Supporting Information: Table S2). Further, 17 lactobacilli species were identified from gut microbiome data of 581 healthy individuals from 11 populations (Supporting Information: Table S3). Using 156 BSH sequences [27] and 6 representative BSH sequences in the KEGG database as a reference (Supporting Information: Table S4), a total of 16 BSH‐encoding lactobacilli species, including *Ligilactobacillus salivarius*, *Ligilactobacillus animalis*, *Limosilactobacillus mucosae*, *Ligilactobacillus ruminis, Limosilactobacillus reuteri*, *Lactiplantibacillus plantarum*, *Lactobacillus amylovorus*, *Ligilactobacillus murinus*, *Limosilactobacillus fermentum*, *Latilactobacillus sakei*, *Lacticaseibacillus casei*, *Lacticaseibacillus rhamnosus*, *Lactobacillus acidophilus*, *Lacticaseibacillus paracasei*, *Lactobacillus gasseri*, and *Lactobacillus delbrueckii*, were obtained from the gut microbiome of healthy individuals (Figure 1D, sorted in descending order of relative abundance). Of the species mentioned above, *L. ruminis* was present in the gut microbiota of most populations (6 out of 11, Supporting Information: Table S5). The most diverse lactobacilli species were observed in samples from Austria (AT; 10 out of 16, Supporting Information: Table S5).

### Classification of lactobacilli species and comparison of deconjugation ability in vitro

**Figure 2:**
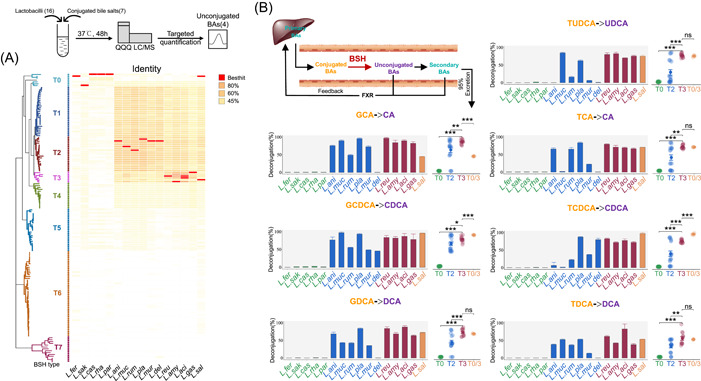
Deconjugation activity of bile salt hydrolase (BSH)‐encoding lactobacilli. (A) Taxonomic characteristics of representative BSH‐encoding lactobacilli strains. Details are shown in Supporting Information: Table S6. (B) In vitro deconjugation activity of representative BSH‐encoding lactobacilli strains. Details are shown in Supporting Information: Table S7. *p < 0.05, **p < 0.01, and ***p < 0.001. In this experiment, unconjugated BAs include CA, CDCA, DCA, and UDCA. DCA and UDCA belong to secondary BAs, which also could transfer from CA and CDCA. CA, Cholic acid; CDCA, Chenodeoxycholic acid; DCA, Deoxycholic acid; GCA, Sodium glycocholate hydrate; GCDCA, sodium glycochenodeoxycholate; GDCA, sodium glycodeoxycholate; TCA, sodium taurocholate hydrate; TCDCA, sodium taurochnodeoxycholate; TDCA, sodium taurodeoxycholate hydrate; TUDCA, sodium tauroursodeoxy cholate; UDCA, Ursodeoxycholic acid.

The 16 representative BSH‐encoding strains of lactobacilli were classified according to the phylogenetic‐based system developed in previous studies [27]. These lactobacilli species mainly encoded three phylotypes of BSH: BSH‐T0, BSH‐T2, and BSH‐T3. *L. fermentum*, *L. sakei*, *L. casei*, *L. rhamnosus*, and *L. paracasei* encoded BSH‐T0. *L. animalis*, *L. mucosae*, *L. ruminis*, *L. plantarum*, *L. murinus*, and *L. delbrueckii* encoded BSH‐T2. *L. reuteri*, *L. amylovorus*, *L. acidophilus*, and *L. gasseri* encoded BSH‐T3. However, in particular, *L. salivarius* encoded both BSH‐T0 and BSH‐T3 (Figure 2A, Supporting Information: Table S6). Thus, the 16 strains of lactobacilli can be classified as Lac (BSH‐T0), Lac (BSH‐T2), Lac (BSH‐T3), and *L. salivarius*.

The deconjugation abilities of the lactobacilli strains were compared by determining the proportion of unconjugated BA product after incubation under anaerobic conditions with conjugated bile salts. Targeted quantitative detection of corresponding unconjugated BAs, including cholic acid (CA), chenodeoxycholic acid (CDCA), deoxycholic acid (DCA), and ursodeoxycholic acid (UDCA), was performed after 48 h hydrolysis (Supporting Information: Figure S3). Compared to other bile salts, the 16 lactobacilli strains generally showed weaker deconjugation ability during the hydrolysis of sodium taurodeoxycholate hydrate (TDCA). Deconjugation abilities of most strains against TDCA were less than 65%, except *L. acidophilus* ATCC 4796 (83.13%; Figure 2B, Supporting Information: Table S7). Apart from TDCA, approximately 80%–100% of the other conjugated bile salts could be converted into unconjugated BAs after incubation with Lac (BSH‐T3) strains, but less than 3% with Lac (BSH‐T0) strains. For the *L. salivarius* BNCC 138618 strain, which encoded both BSH‐T0 and BSH‐T3, only deconjugations of GCA, GCDCA, and TCDCA were significantly different from the Lac (BSH‐T3) strains (scatter plots of Figure 2B, Supporting Information: Table S7). In general, the deconjugation ability of the lactobacilli strains could be represented as follows: Lac (BSH‐T3) ≈ *L. salivarius* > Lac (BSH‐T2) > Lac (BSH‐T0) (scatter plots of Figure 2B).

Interestingly, strains classified as Lac (BSH‐T2) showed substrate preferences. For instance, *L. delbrueckii* subsp. *bulgaricus* ATCC 11842 could effectively hydrolyze sodium glycochenodeoxycholate (GCDCA; 45.64%) and sodium taurochnodeoxycholate (TCDCA; 79.85%), while the deconjugation abilities for other bile salts were less than 1% (bar plots in Figure 2B, Supporting Information: Table S7). *L. mucosae* DSM 13345 showed higher deconjugation ability for sodium glycocholate hydrate (GCA; 89.76%), GCDCA (97.34%), and sodium tauroursodeoxy cholate (TUDCA; 84.46%), but lower deconjugation ability for sodium taurocholate hydrate (TCA; 2.18%) and TCDCA (1.83%; bar plots in Figure 2B, Supporting Information: Table S7).

### Administration of lactobacilli alters the gut microbiome of mice

In a bid to further explore the effect of lactobacilli in vivo, four lactobacilli strains including *L. fermentum* BNCC 194390 encoding BSH‐T0, *L. animalis* BNCC 134981 encoding BSH‐T2, *L. reuteri* ATCC 27755 encoding BSH‐T3, and *L. salivarius* BNCC 138618 encoding both BSH‐T0 and BSH‐T3 (species with the highest relative abundance in each lactobacilli category) were administered to male C57BL/6J mice for 2 weeks.

**Figure 3:**
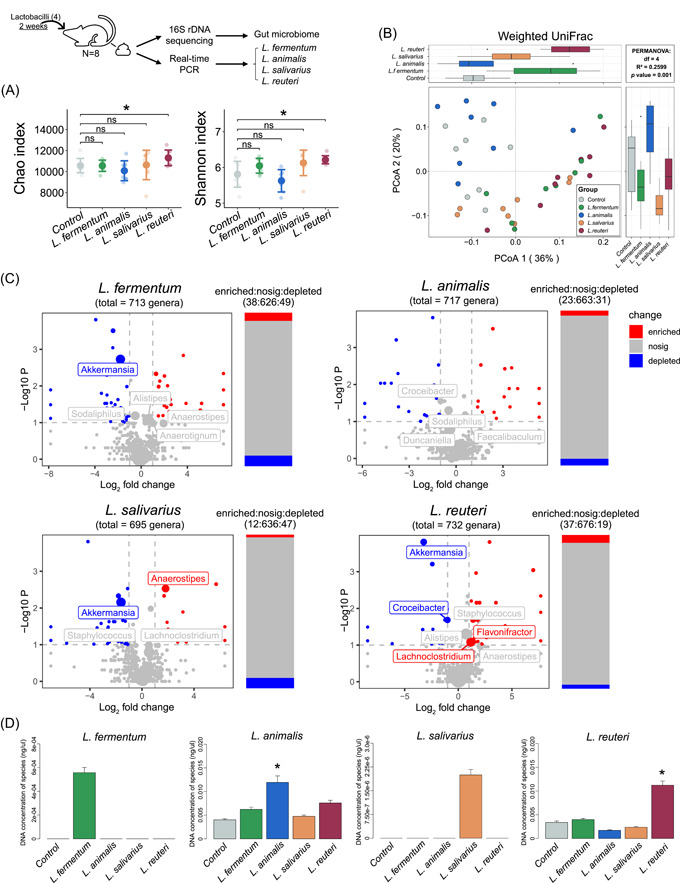
Treatment with lactobacilli alters the structure of the fecal microbiome in mice. (A) Alpha diversity (Chao richness and Shannon diversity index) of the fecal microbiome. (B) Two‐dimensional PCoA of the fecal microbiome based on WUniFrac. PERMANOVA was used for statistical significance of beta diversity; *p* < 0.05 was considered as statistically significant. (C) Volcano plots illustrate the genus enrichment analysis of the fecal microbiome. The Wilcoxon rank sum test was used for analysis. The labels represent the top 15 genera, and the sizes of labels and points were proportional to the average abundance of genera in each group. (D) Concentrations of four lactobacilli species (including *Limosilactobacillus fermentum*, *Ligilactobacillus animalis*, *Ligilactobacillus salivarius*, and *Limosilactobacillus reuteri*) in the fecal microbiota of mice in different groups. PCoA, principal coordinates analysis. *p < 0.05, **p < 0.01, and ***p < 0.001.

The 16S rDNA sequencing was performed using mouse fecal genomic DNA two weeks after administration. Compared with the control group, only the *L. reuteri*‐administered group showed a significantly higher level of alpha diversity (Wilcoxon rank‐sum test; *p* < 0.05, Figure 3A). Amplicon sequencing data also revealed distinct clusters of microbial structures in each group (PERMANOVA with Weighted Unifrac distance; *R* ^2^ = 0.2599, *p* = 0.001). Except for the *L. animalis‐*administered group, other lactobacilli‐administered groups were found to be significantly different from the control group (Figure 3B). Moreover, the ratio of *Bacteroidetes* to *Firmicutes* (B:F) was decreased in all the lactobacilli‐administered groups compared to the control group (B:F = 3.4), with the *L. reuteri*‐administered group showing the greatest decrease (B:F = 2.2, Supporting Information: Figure S4A).

Through absolute quantitative analysis (described in the Materials and Methods section), the *L. reuteri*‐administered group was found to show the most highly enriched and the least depleted genera (|log_2_(fold change)| > 1, −log_10_(*p* value) > 1). Notably, among the top 15 genera, both *Lachnoclostridium* (average abundance = 0.0699; log_2_(fold change) = 1.17) and *Flavonifractor* (average abundance = 0.0180; log_2_(fold change) = 1.05) were found to be enriched in the *L. reuteri*‐administered group (Figure 3C). Also, a significant increase in the relative abundance of *Alistipes* and *Staphylococcus* was observed in the *L. reuteri*‐administered group (Supporting Information: Figure S4B). Furthermore, in comparison with the control group, a significant decrease in the abundance of *Akkermansia* was noted in all the lactobacilli‐administered groups, except for the *L. animalis*‐administered group. Thus, treatment with BSH‐encoding lactobacilli species, especially *L. reuteri*, altered the structure of the gut microbiome in mice.

The abundance of the *Lactobacillus* genus did not differ significantly among the groups in spite of lactobacilli administration (Supporting Information: Figure S4B). Therefore, specific primers of the four lactobacilli species were designed for targeted quantification in mice feces (Supporting Information: Figure S5, Table S8). Compared with the control group, the relative abundance of *L. animalis* and *L. reuteri* was significantly increased (Figure 3D). Since *L. fermentum* and *L. salivarius* are not indigenous to the gut microbiota of mice, no signal was observed in any of the groups, except for the group administered these two lactobacilli strains (Figure 3D). The above results showed that administration of the four lactobacilli strains significantly increased the concentrations of their respective species in mice.

### Administration of lactobacilli alters serum and fecal metabolomes of mice

**Figure 4:**
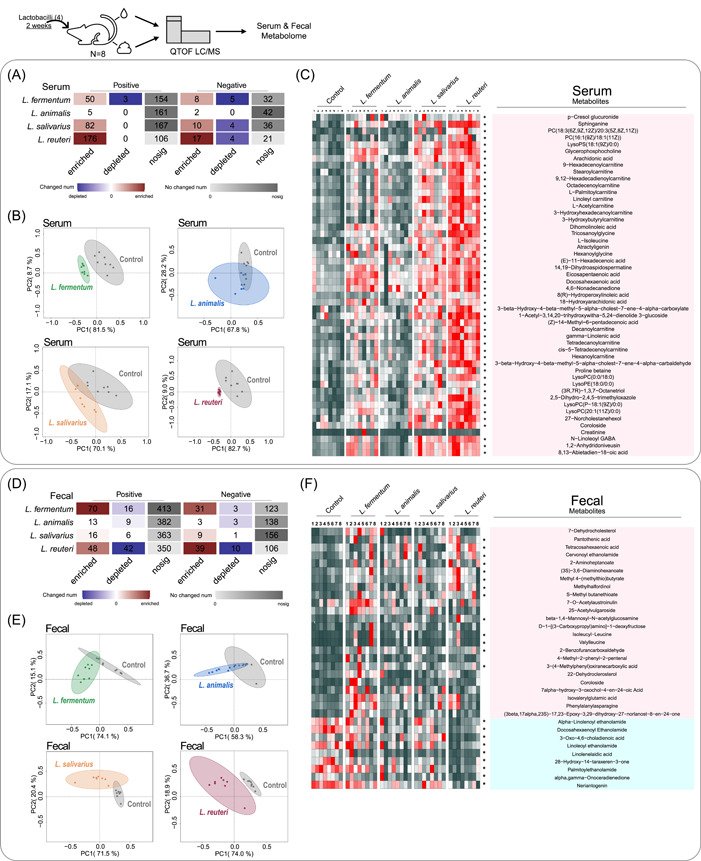
Treatment with lactobacilli alters the serum and fecal metabolomes of mice. (A) Heatmap of differential metabolic ion enrichment analysis in serum. The red color shows that the relative concentration of the feature was higher in the lactobacilli‐administered group, while the blue color shows that the relative concentration of the feature was higher in the control group; the gray color shows that the relative concentration of the feature did not differ between the two groups. (B) Score plots of principal component analysis based on the differential metabolites (VIP > 1, *pFDR* ≤ 0.05) in serum. (C) Heatmap of the differential metabolites (*pFDR* ≤ 0.05) in serum. (D) Heatmap of differential metabolic ion enrichment analysis in feces. (E) Score plots of principal component analysis based on the differential metabolites (VIP > 1, *pFDR* ≤ 0.05) in feces. (F) Heatmap of the differential metabolites (*pFDR* ≤ 0.05) in feces. Asterisks represent differential metabolites in the *L. reuteri*‐administered group. Positively charged ions are collected in ESI+ mode, and negatively charged ions are collected in ESI− mode.

We then investigated the effects of the administration of lactobacilli on the metabolomes of mice. A total of 573 and 1630 features were detected in the serum and feces, respectively. Analysis of the total ion chromatograms obtained using serum samples showed that the *L. reuteri*‐administered group showed the highest number of differential features compared to the control group (Figure 4A, Supporting Information: Table S9). Principal component analysis of differential features in each group showed that the serum metabolites of mice in the *L. fermentum* and *L. reuteri* administration groups were substantially different from those in the control group (Figure 4B). Notably, lactobacilli treatment altered 50 metabolites (upregulated) in serum, while the administration of *L. reuteri* resulted in 49 metabolite changes (Figure 4C, Supporting Information: Table S10). The upregulated metabolites were mainly lipids and lipid‐like molecules, including 27−Norcholestanehexol (an intermediate of cholesterol metabolism), and some carnitine metabolites, such as L‐acetylcarnitine (Supporting Information: Table S10). In feces, again, the *L. reuteri*‐administered group still had the highest number of differential features and was the only group substantially different from the control group (Figure 4D,E, Supporting Information: Table S9). In addition, lactobacilli treatment altered 33 metabolites in feces (24 upregulated and 9 downregulated), while the *L. reuteri*‐administered group showed 22 significant metabolites (13 upregulated and 9 downregulated) (Figure 4F, Supporting Information: Table S10). The differential metabolites were mainly lipid molecules, organic acids, and derivatives. The downregulated metabolites included some steroid derivatives, such as 3‐oxo‐4, 6‐cholionic acid (Supporting Information: Table S10). These results suggest that *L. reuteri* administration had significant effects on the serum and fecal metabolomes of mice, especially in promoting lipid and cholesterol metabolism, which could have potentially played a role in preventing fat accumulation.

### Regulatory effect of lactobacilli species on enterohepatic BA pools in mice

**Figure 5:**
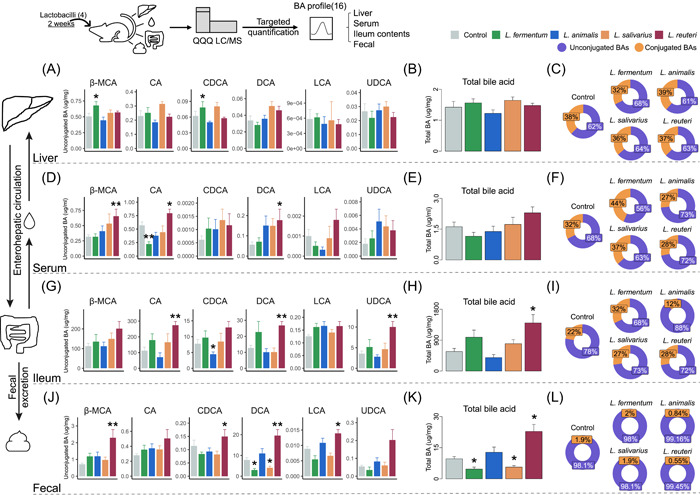
Treatment with lactobacilli alters the composition of the bile acid (BA) pool in mice. (A) Unconjugated BAs. (B) Total BA. (C) Proportion of unconjugated BAs and conjugated BAs in the liver of mice from different groups. (D) Unconjugated BAs. (E) Total BA. (F) Proportion of unconjugated BAs and conjugated BAs in the serum of mice from different groups. (G) Unconjugated BAs. (H) Total BA. (I) Proportion of unconjugated BAs and conjugated BAs in the distal ileum of mice from different groups. (J) Unconjugated BAs. (K) Total BA. (L) Proportion of unconjugated BAs and conjugated BAs in the fecal samples of mice from different groups. Statistically significant differences between the two groups were determined using a paired *t* test. *p < 0.05, **p < 0.01, and ***p < 0.001. Unconjugated BAs include CA, CDCA, DCA, and UDCA. Conjugated BAs include GCA, GCDCA, GDCA, TCA, TCDCA, TDCA, and TUDCA. Total BAs are the sum of all the detected BAs (including unconjugated BAs and conjugated BAs). CA, cholic acid; CDCA, chenodeoxycholic acid; DCA, deoxycholic acid; F, female; LCA, lithocholic acid; M, male; UDCA, ursodeoxycholic acid; β‐MCA, β‐Muricholic acid.

To determine the effect of the lactobacilli species encoding different BSH phylotypes on enterohepatic BA profiles, the targeted quantification of BAs was performed in the liver, blood, ileum, and feces of mice (Supporting Information: Figures S6–S9, Table S11). The results showed that minor changes were observed in the hepatic BA pool, with only the *L. fermentum*‐administered group showing an increase in the concentration of β‐muricholic acid (β‐MCA) and CDCA (Figure 5A, Supporting Information: Figure S10A). Neither the total BA levels nor the unconjugated/conjugated BA ratio was found to be significantly different in the liver (Figure 5B,C). In serum, treatment with *L. reuteri* resulted in a significant increase in the concentrations of β‐MCA, CA, and DCA, indicating an upward trend in the total BA level and the percentage of unconjugated BA (Figure 5D–F). In the distal ileum and fecal excretion, both the BA profile and the total BA levels (Figure 5H,K) changed significantly after the administration of *L. reuteri*. Specifically, the concentrations of CA, DCA, and UDCA in the distal ileum and fecal excretion of β‐MCA, CDCA, DCA, and lithocholic acid (LCA) were all significantly increased in the *L. reuteri*‐administered group (Figure 5G,J). However, the unconjugated/conjugated BA ratio in the feces was increased (Figure 5L), but decreased in the distal ileum (Figure 5I) of the *L. reuteri‐*administered group, which might be attributed to the increased levels of conjugated BAs (Supporting Information: Figure S10). These results suggested that lactobacilli strains were pleiotropic regulators of enterohepatic BA metabolism. The significant increase of unconjugated BA levels and changes of the BA profile in *L. reuteri*‐administered mice further confirmed that the BSH‐T3‐encoding lactobacilli species is an important regulator of BA metabolism.

## DISCUSSION

Since BSHs clearly play an important role in the regulation of host BA metabolism and physiological response, it is necessary to evaluate the distribution, abundance, and function of BSHs. A previous study used function‐driven metagenomics to identify BSHs in microbial communities for the first time and clustered them based on environmental origin, but the sample size was small [32]. Our previous work revealed the taxonomic and abundance profiling of BSHs based on large amount of human gut microbiome and assessed BSH activity by classifying the target sequence into specific phylotypes [27]. Based on previous work, this study, which comprehensively assessed BSH‐encoding gut bacteria through multivariate analysis and data mining of extensive metagenome data, discovered crucial BSH‐encoding lactobacilli species. Compared with other BSH‐encoding genera, lactobacilli was the most reported (Supporting Information: Figure S11, Table S12), including *L. gasseri* [22], *L. reuteri* [33], *L. salivarius* [34], and so on. A previous study reported about 39 BSH‐encoding lactobacilli species, but including some species that are not indigenous to the gut, such as *L. agilis* and *L. vaginalis*, which are typically found in the saliva and the vagina, respectively [30]. Using healthy human gut metagenomes, 16 BSH‐encoding lactobacilli species were systematically identified and classified according to the BSH phylotypes. As mentioned, the genus *Lactobacillus* was reclassified based on core genome phylogeny, pairwise average amino acid identity, clade‐specific signature genes, physiological criteria, and the ecology of the organisms [31]. The classification of lactobacilli based on BSH phylotype does not seem to be sufficiently relevant to the new taxonomy. Specifically, although three *Lacticaseibacillus* strains encoded BSH‐T0, *Limosilactobacillus fermentum* and *L. reuteri* encoded BSH‐T0 and BSH‐T3, respectively. *Ligilactobacillus animalis*, *Ligilactobacillus ruminis*, and *Ligilactobacillus murinus* belong to Lac (BSH‐T2), while *L. salivarius* does not encode BSH‐T2 (Figure 2A).

To examine the BSH activities of lactobacilli strains in vitro, Jimmy et al. [35] compared the deconjugation activities of five lactobacilli against four BAs and found that *L. plantarum* DGIA1 showed excellent deconjugation abilities. In contrast, our study systematically compared the deconjugation activities of 16 BSH‐phylotype classified lactobacilli strains against seven conjugated BAs and found that the lactobacilli strains that encoded BSH‐T3, including *L. reuteri* ATCC 27755, *L. amylovorus* BNCC 135321, *L. acidophilus* ATCC 4796, and *L. gasseri* BNCC 135057, had the highest deconjugation activities. However, it is noteworthy that some BSH‐T2‐encoding strains showed bile salt substrate preferences (Figure 2B, Supporting Information: Table S7). Similarly, Matthew et al. [36] also observed that BSHs encoded by *Lactobacillus acidophilus* NCK1909 and *Lactobacillus gasseri* NCK2253 showed substrate preferences, and might affect the growth of the strains. Then, they recently structurally identified a loop that predicts BSH preferences for either glycine or taurine substrates; specifically, taurine‐preferring enzymes exclusively contained Gly‐Val/Thr‐Gly motifs, while glycine‐preferring enzymes exclusively contained Ser‐Arg‐Gly/Ser motifs [37]. A review summarized the reported substrate‐specific BSHs and classified them according to substrate preference, and explored key residues and secondary structural elements that were potentially involved in determining substrate preferences [29]. Another study found that *Lactobacillus salivarius* showed a preference for substrates with a taurine head mainly because of the dehydroxylated sterol ring for hydrolysis and suggested the importance of Asp19, Asn79, Asn171, Arg224, Gln257, Asn262, and Glu270 for binding of the substrate amino acid head [38]. However, the substrate preference of BSH‐T2‐encoding lactobacilli in this study is more complex, which could not be completely divided into glycine‐preferring and taurine‐preferring lactobacilli. We speculate that these properties may be related to the structures of different BSH‐T2, and further in‐depth studies with structural analysis of BSH‐T2 with specific substrates along with comprehensive amino acid substitution mutagenesis are needed to understand the basis for the substrate preferences.

Compared with humans, the gut bacteria of mice can produce more diverse secondary BAs [39]. Moreover, glycine amidation is predominant in humans, while taurine amidation is predominant in mice [40], which corresponds to the low concentration of enterohepatic glycine‐BAs in our results (Figure 5, Supporting Information: Figure S10). Although the composition of gut microbiota and BAs varies greatly between the model animals and humans, in vivo research in mice remains indispensable in this field (Supporting Information: Figure S12, Table S13). However, most of the studies focused on demonstrating the regulation of a single lactobacilli strain on BA metabolism [41, 42]. For example, *L. plantarum* CCFM8661 has been reported to considerably increase hepatic BA synthesis, bile and biliary glutathione output, and fecal BA excretion in mice [43]. *L. rhamnosus* GG supplementation inhibits BA de novo synthesis, decreases hepatic BA, and increases BA excretion [42]. Our study examined the impact of four lactobacilli strains that encoded different BSH phylotypes on the enterohepatic BA profile in mice and found that administration of BSH‐T3‐encoding *L. reuteri* ATCC 27755 significantly changed the structure of gut microbiomes and metabolomes in mice, showing excellent ability to change the enterohepatic BA profile and increase BA excretion. In addition, several studies have shown that high fat intake may trigger chronic disease by altering the BA profile [44]. Analysis of the metabolome in mice revealed that administration of BSH‐encoding lactobacilli altered a number of metabolites associated with lipid and sterol metabolism (Figure 4), suggesting a possible beneficial effect in metabolic diseases associated with a high‐fat diet.

BSHs paralogs were reported in our previous study [27]; *L. fermentum* MTCC 8711 was reported to encode two *bsh* genes [45] and *L. plantarum* WCFS1 was reported to encode four *bsh* genes [46]. Some lactobacilli strains in this study also encoded more than one *bsh* gene, including *L. acidophilus* ATCC 4796, which encoded two genes belonging to BSH‐T3, *L. amylovorus* BNCC 135321, which encoded three genes belonging to BSH‐T3, *L. paracasei* BNCC 189800, which encoded two genes belonging to BSH‐T0, and *L. salivarius* BNCC 138618, which encoded genes belonging to BSH‐T0 and BSH‐T3, respectively (Supporting Information: Table S6). To the best of our knowledge, few studies have investigated the effect of bacteria with BSH paralogs on the BAs' metabolic function. Interestingly, our findings showed that though *L. salivarius* BNCC 138618 had a comparable deconjugation ability to Lac (BSH‐T3) strains in vitro, the treatment had no significant effect on enterohepatic BA pools in an in vivo study; thus, further mechanistic studies are needed.

The following are the main drawbacks of this study: (1) the significance of the lactobacilli genus and selected species might be biased by the public human gut metagenomic data used and (2) the mechanism(s) by which the lactobacilli strains regulate BA metabolism should be explored further using appropriate in vitro and in vivo models. Collection of metagenomic data will be continued to obtain superior data sets for further genomic analysis, and attempts will be made to construct *bsh*‐knockout lactobacilli and animal models of BA disorders to explore the clinical application possibilities of lactobacilli in disease therapeutics.

## CONCLUSION

A phylogenetic‐based system to classify 156 BSHs into eight phylotypes was provided previously. In this study, we identified lactobacilli as the most important BSH‐encoding genus and evaluated the deconjugation abilities of 16 lactobacilli species on BAs in vitro and in vivo. BSH‐T3‐encoding lactobacilli were found to play an important role in modulating the enterohepatic BA profile. This study, combined with our previous work, showed the importance of BSHs' classification. While the exact mechanisms by which bacteria regulate BAs' metabolism in disease progression and clinical trials of lactobacilli as adjuvant therapy remain to be further explored, the discovery of BSH‐coding bacteria might still aid in identifying strains with therapeutic potential in related diseases.

## MATERIALS AND METHODS

### Sequence data collection

Lactobacilli genome sequences were downloaded from the Ensembl database (https://asia.ensembl.org/). Reference genomes were obtained from the HMP database (https://www.hmpdacc.org/) to predict and classify the genes and proteins in the lactobacilli genomes [27, 47]. The individual metagenomic sequence data refer to a data set constructed in previous studies that included the data of 581 healthy individuals [27] from 11 populations in six continents, including the Hadza ethnic group of Tanzania (HZ) [48] of Africa, China (CN) [49, 50], Japan (JP) [51], and South Korea (KR) [52] of Asia, Austria (AT) [53], Denmark (DK) [54], France (FR) [55], and Sweden (SE) [56] of Europe, Australia (AU, PRJEB6092) [57] of Oceania, the United States (US) [58] of North America (N‐America), and Peru (PE) [59]of South America (S‐America). Pairwise amino acid sequence alignments were performed using BLASTP (v 2.2.29+) [60].

### Phylogenetic tree

BSH protein sequences were aligned using ClustalW, and phylogenetic trees were built using the maximum likelihood method (Jones–Taylor–Thornton model) in MEGA software (v7.0). Interactive Tree Of Life (iTOL) (https://itoL.embL.de/) was used to import the data on the abundance of 156 BSH sequences into the phylogenetic tree and fill in the colors as needed. A dendroscope (v3.4.7) was used to embellish the phylogenetic tree by adjusting of labels and filling of colors.

### Materials and bacterial strains

Sodium glycocholate hydrate (CAS: 863‐57‐0), sodium glycochenodeoxycholate (CAS: 16564‐43‐5), sodium glycodeoxycholate (CAS: 16409‐34‐0), sodium taurocholate hydrate (CAS: 345909‐26‐4), sodium taurochnodeoxycholate (CAS: 6009‐98‐9), sodium taurodeoxycholate hydrate (CAS: 207737‐97‐1), and sodium tauroursodeoxy cholate (CAS: 35807‐85‐3) were purchased (Yuanye Bio‐Technology Co, Ltd) to perform the targeted quantification of deconjugated lactobacilli species in vitro. Standards of GCA, GCDCA, GDCA, GLCA, GUDCA, TCA, TCDCA, TDCA, TLCA, TUDCA, CA, CDCA, DCA, LCA, UDCA, and β‐MCA and their corresponding isotopic standards were purchased (ZZBIO Co, Ltd) for quantitative assays. 16 lactobacilli strains were used in BA deconjugation assays (Supporting Information: Table S6).

### Conjugated bile salt deconjugation using lactobacilli strains

Lactobacilli strains were diluted to pre‐log phase (OD_600nm_ = 0.1, 10^7^ CFU) levels in a freshly prepared MRS medium. Stock solutions of conjugated bile salts were added to each culture to obtain a final concentration of 4 μM for each bile salt. In line with previous work [23], we monitored the growth of 16 lactobacilli strains and confirmed that all strains reached the stationary phase of growth after 48 h. Then, cultures were incubated in an anaerobic chamber at 37°C for 48 h.

### Animal study

Eight‐week‐old C57BL/6J male mice (20–22 g) were obtained from GemPharmatech Co, Ltd. Mice were allowed to acclimatize to the environment and provided free access to food and water for at least 1 week before the experiment, and then randomly distributed into different groups and fed a standard chow diet (Jiangsu Xietong Medical Bioengineering Co, Ltd). Lactobacilli cultures were collected after an incubation period of 48 h, washed, and diluted with sterile phosphate‐buffered saline (PBS) to 5 × 10^9^ colony‐forming units/mL for gavage administration (200 μL per mice/day). There were initially 10 mice in each group. Given the considerable variations in the metabolism of individual mice, the mice with the highest and lowest total BA concentrations in each group were excluded, and these samples were also excluded from the analysis of the gut microbiome and metabolome. The group sizes were selected according to minimal experimental requirements.

### 16S rDNA amplicon sequencing and data analysis

Stool samples were collected from each mouse after 2 weeks of daily lactobacilli administration. Genomic DNA was extracted using a DNA kit (TIANGEN Biotech Co, Ltd). DNA (1%) from an exogenous strain (*Sporosarcina pasteurii* ATCC 11859) was added to perform the absolute quantification of bacterial changes. Then, a mixture of DNA (30 to 40 ng) was used to generate amplicons. The V3 and V4 hypervariable regions (F: 5′‐ACTCCTACGGGAGGCAGCA‐3′; R: 5′‐GGACTACHVGGGTWTCTAAT‐3′) of prokaryotic 16S rDNA were selected for the generation of amplicons and subsequent taxonomic analysis. DNA libraries were multiplexed and loaded onto an Illumina NovaSeq instrument according to the manufacturer's instructions (Illumina).

Sequencing was performed using a paired‐end configuration, external standard strains were identified, and absolute abundance levels were calculated. Sequence data analyses were mainly performed using the QIIME 2. Statistical analysis was performed using R software (v3.3.2). To compare the richness and evenness of the genera among samples, the alpha diversity values between different groups were visualized using the Chao [61] and Shannon index [62]. Beta diversity was measured based on weighted UniFrac (WUniFrac) distance matrices using principal coordinates analysis in the vegan package. Enrichment analysis of genera was conducted based on the absolute abundance of total genera. Enriched genera were screened using the following criterion: log_2_(fold change) > 1 and −log_10_(*p* value) > 1. Similarly, the depleted genera were screened using the following criterion: log_2_(fold change) < −1 and −log_10_(*p* value) > 1.

### Quantification of lactobacilli species in mice

The AceQ qPCR SYBR Green Master Mix (Vazyme Biotech Co, Ltd) was used to determine the amounts of lactobacilli species by real‐time PCR. The set of primers used for the amplification of bacteria is shown in Supporting Information: Table S8. Standard curves were constructed using the diluted DNA of corresponding lactobacilli strains.

### Measurement of BAs

An Agilent 1290 Infinity liquid chromatograph (LC) system coupled to an Agilent 6470 triple quadrupole (QQQ) mass spectrometer (MS) was used to quantify BAs in bacterial culture and the serum, liver, ileum content, and feces of mice. These included GCA, GCDCA, GDCA, GLCA, GUDCA, TCA, TCDCA, TDCA, TLCA, TUDCA, CA, CDCA, DCA, LCA, UDCA, and β‐MCA (only in mice). CA‐d4, GCDCA‐d4, GDCA‐d4, GLCA‐d4, GUDCA‐d4, TCA‐d4, TCDCA‐d4, TDCA‐d4, TLCA‐d4, TUDCA‐d4, CA‐d4, CDCA‐d4, DCA‐d4, LCA‐d4, UDCA‐d4, and β‐MCA‐d5 were used as internal standards. The detection of BAs was performed in the negative ion mode by multiple reaction monitoring modes. Agilent MassHunter software (version B.08.00) was used for instrumental control and data acquisition. It should be noted that quantification of GLCA was not performed in mouse serum and liver because its concentrations were below the detection limits. The methods for sample preparation and detailed chromatographic conditions are described in the Supporting Information.

### Metabolomics

Untargeted analyses were performed using an Agilent 1290 Infinity LC system coupled to an Agilent 6545 quadrupole time‐of‐flight (Q‐TOF) MS equipped with an electrospray ionization source operating in both positive and negative ion modes. The serum and feces used for untargeted metabolomics analysis were processed in a manner similar to that for the targeted analysis, except that the isotope standard working solutions were replaced with 0.1 μg/mL L‐2‐chlorophenylalanine and 1 μg/mL ketoprofen and pooled. QC samples were obtained from the pooled aliquots of each serum and fecal homogenate liquid sample and pretreated using the same procedure. Agilent MassHunter software (version B.08.00) was used for instrumental control and data acquisition, and the XCMS package of the R program was run for data pretreatment, including peak detection discrimination, baseline correction, and nonlinear retention time alignment. Identification of differential metabolite signatures was performed based on the accurate mass and MS/MS fragments by searching through the Human Metabolome Database. Some of them were unambiguously confirmed with available reference compounds. The detailed chromatographic conditions are described in the Supporting Information.

### Statistical analysis

All values were expressed as mean ± SEM. Statistically significant differences were detected between the two groups using the Wilcoxon rank‐sum test, followed by false discovery rate correction. The correlation analysis was done with Spearman rank correlations. Multiple comparisons were performed by multivariable‐adjusted analysis using the linear regression model. All analyses were performed using R (v3.3.2), and *p* < 0.05 was considered to be statistically significant. The data were visualized by ImageGP [63]. Supervised orthogonal partial least‐squares discriminant analysis (OPLS‐DA) was applied to identify the differences in the metabolic phenotypes between groups. Those metabolic features with an adjusted *p* value (*p*FDR) < 0.05 and variable importance in the projection (VIP) value > 1.0 in the OPLS‐DA model were defined as differential metabolites.

## AUTHOR CONTRIBUTIONS

Ping Li and Jing Li conceived and designed the study. Ziwei Song performed experiments and analyzed the data. Shuo Feng collected the metagenome data and performed the analyses. Xingchen Zhou analyzed the 16S rDNA sequencing data. Zhengxing Song analyzed the untargeted metabolomics data. Ping Li, Jing Li, and Ziwei Song interpreted the data and wrote the manuscript. All authors read and approved the final manuscript.

## CONFLICT OF INTEREST STATEMENT

The authors declare no conflict of interest.

## ETHICS STATEMENT

Experiments involving animals were conducted in accordance with the Guidelines for Animal Experimentation at China Pharmaceutical University (Nanjing, China), and the protocols were approved by the Animal Ethics Committee (No. 2021‐09‐023).

## Supporting information

Supporting information.

Supporting information.

## Data Availability

(For all public metagenomic data used in this manuscript, the web links or references are provided in the Materials and Methods Section. Other data generated in this manuscript are included in additional files. For all public metagenomics data used in this manuscript, web links or references are provided. All codes used are saved in GitHub https://github.com/chaunceyZZ/iMeta2023TaxonomicIdentificationofBileSaltHydrolase-EncodingLactobacilli.git). Supporting Information (figures, tables, graphical abstract, and methods) may be found in the online DOI or iMeta Science http://www.imeta.science/.
