# Supplementary material for: Taxonomic identification of bile salt hydrolase‐encoding lactobacilli: Modulation of the enterohepatic bile acid profile
Source: Imeta. 2023 Jul 16;2(3):e128. doi: 10.1002/imt2.128 (PMC10989828; doi:10.1002/imt2.128)
Supplement: Supplementary file 1 — Supporting information. [file IMT2-2-e128-s002.docx]

**Supporting Information to:**

**Taxonomic identification of bile salt hydrolase-encoding lactobacilli: modulate enterohepatic bile acid profile**

**Running title**: Characterization of lactobacilli modulating enterohepatic bile acids profile

Ziwei Song ^1#^, Shuo Feng ^2#^, Xingchen Zhou ^3^, Zhengxing Song ^2^, Jing Li ^1,2*^, Ping Li ^1*^

^1^ State Key Laboratory of Natural Medicines, China Pharmaceutical University, Nanjing 210009, China.

^2^ School of Life Science and Technology, China Pharmaceutical University, Nanjing 210009, China.

^3^ Beijing Key Laboratory of New Molecular Diagnosis Technologies for Infectious Disease, Department of Biotechnology, Beijing Institute of Radiation Medicine, Beijing 100850, China.

# These authors contributed equally: Ziwei Song, Shuo Feng.

^*^ Correspondence: [liping2004@126.com](mailto:liping2004@126.com) (Ping Li), [lj_cpu@126.com](mailto:lj_cpu@126.com) (Jing Li).

**Supplementary Methods**

**Sample preparation for LC-MS/MS**

For targeted quantitative analysis using bacteria, a mixture containing 180 μL of each bacterial culture and 20 μL of corresponding standard working solution (CA-d4, CDCA-d4, DCA-d4, UDCA-d4, with a final concentration of 4 μM) was acidified to a pH = 1.0 using 200 μL of 6N HCl. The culture was then extracted using 600 μL of ethyl acetate. In the case of an emulsion, the biphasic solution was centrifuged at 13,000 rpm for 10 min to obtain a 400 μL of clear separations and dried with nitrogen. Finally, the residue was reconstituted in 100 μL of 50% aqueous acetonitrile solution, and 1 μL of this solution was used for LC/MS analysis.

For targeted quantitative analysis using blood from mice, a 30 μL aliquot of serum was mixed with 10 μL of internal standard working solution (Table S11). Then, an 80 μL aliquot of methanol solution was added and vortexed for 2 min to extract the BAs. After centrifugation for 10 min at 13,000 rpm, 80 μL of supernatant was carefully transferred into a separate tube and dried with nitrogen. Finally, the residue was reconstituted in 100 μL of a 50% aqueous acetonitrile solution, and 10 μL of this solution was used for LC/MS analysis.

For targeted quantitative analysis using other tissues from mice, 10 mg of liver, 0.4 mg of freeze-dried ileum contents, 4 mg of freeze-dried feces from mice were each added to 100 μL of ultrapure water and ultrasonically pulverized for 5 min; 100 µL slurry was mixed with 10 μL of a working solution of internal standards (Table S11). Then, 490 μL aliquots of methanol were added and ultrasonically pulverized was performed for 5 min to extract the BAs. After centrifugation for 10 min at 13000 rpm, 500 μL of supernatant was carefully transferred into another tube and dried with nitrogen. Finally, the residue was reconstituted in 100 μL of 50% aqueous acetonitrile solution, and centrifuged for 10 min at 13000 rpm; 80 μL of supernatant was carefully transferred into another tube and 1 μL was used for LC/MS analysis.

**LC-MS/MS conditions**

The analytical column used for untargeted analyses was a Waters HSS T3 (2.1 x 100 mm inner diameter, 1.8 μm particle size, Waters Corporation, Wexford, Ireland) with a flow rate of 0.4 mL/min at 50 ℃. The mobile phase used for the ESI+ mode consisted of 0.1% aqueous formic acid (A) and acetonitrile (B). For the ESI- mode, the mobile phase consisted of (A) 10 mM ammonium acetate aqueous solution and (B) 10 mM ammonium acetate water/acetonitrile (1:9) solution. A linear gradient elution was optimized as follows: 0-1 min, 5% B; 1-3 min, 5%-15% B; 3-5 min, 15%-65% B; 5-9 min, 65%-85% B; 9-10 min, 85%-100% B; 100% B for 2 min; then, the initial conditions were applied again for 3 min, to achieve equilibration. The drying gas temperature was set at 300 ℃, drying gas flow rate was set at 8 L/min, nebulizer gas was set at 35 psi, fragmental voltage was set at 120 V, and capillary voltage was set at 3500 V. A full scan was acquired at 50 to 1050 m/z for each sample under the high-resolution mode (Extended Dynamic Range 2GHz). Reference masses were introduced at m/z 112.9855 and 980.0163 for accurate mass calibration.

The analytical column used for targeted quantitative analysis was a Waters HSS T3 (2.1 mm × 100 mm inner diameter, 1.8 μm particle size, Waters Corporation, Wexford, Ireland) with a flow rate of 0.4 mL/min at 45 ℃. Water and acetonitrile both containing 0.1% formic acid were used as mobile phases A and B, respectively. The gradient elution program was as follows: 5%-25% B at 0-1 min, 25%-30% B at 1-9 min, 30%-40% B at 9-10 min, 40%-45% B at 10-17 min, 45%-95% B at 17-18.5 min, and 95% B for 2 min; then, the initial conditions were applied for 3 min, to achieve equilibration. The nebulizing gas flow was set at 3 L/min, the heating gas flow was set at 10 L/min, the drying gas flow was set at 10 L/min, the interface temperature was set at 300 °C; the DL temperature was set at 250 °C; and the heat block temperature was set at 400 °C. A total of 16 BAs were quantitatively measured based on a stable isotope-labeled internal standard calibration strategy. All the standard curves showed good linearity with regression coefficients r^2^ > 0.99 (details were summarized in Figure S7-10).

**Supplementary Figures**

**Figure S1. Phylogenetic tree and relative abundances of 156 BSHs in human microbiome.** The same color of the column represents bile salt hydrolase (BSH) from the same genus. The lighter the color of the column, the lower the proportion of the total relative abundance of BSHs encoded by the genus in the phylotype. Genera with abundances less than 5% are represented by grey columns.

**Figure S2. Radar analysis of representative BSH-encoding genera.**

**Figure S3. Growth curve of 16 lactobacilli strains.**

**Figure S4. Administration of lactobacilli alters the composition of the gut microbiota of mice.** (A) Taxonomic analysis of the top 5 phyla of gut microbiota among different groups; (B) Relative abundance of gut bacteria at the genus level among different groups.

**Figure S5. Standard curve of four lactobacilli species generated from RT-PCR.**

**Figure S6. Standard curve of bile acids in mice liver from liquid chromatography triple quadrupole mass spectrometry.**

**Figure S7. Standard curve of bile acids in mice serum from liquid chromatography triple quadrupole mass spectrometry.**

**Figure S8. Standard curve of bile acids in mice ileum from liquid chromatography triple quadrupole mass spectrometry.**

**Figure S9. Standard curve of bile acids in mice feces from liquid chromatography triple quadrupole mass spectrometry.**

**Figure S10. Conjugated bile acids in enterohepatic circulation** **of mice in different groups.** (A) liver; (B) serum; (C) ileum; (D) feces.

**Figure S11. Bacteria studies in published research articles on bile acid and bile salt hydrolase (Web of science, 28/7/2022).** Details are shown in Table S12.

**Figure S12. Experimental models used in published research articles on the gut microbiota and bile acid (Web of science, 28/7/2022).** Details are shown in Table S13.
